# Vascular memory: can we broaden the concept of the metabolic memory?

**DOI:** 10.1186/1475-2840-11-44

**Published:** 2012-07-12

**Authors:** György Jermendy

**Affiliations:** 1 Teaching Department of Internal Medicine, Bajcsy-Zsilinszky Hospital, Maglódi út, 89-91, 1106, Budapest, Hungary

**Keywords:** Metabolic memory, Metabolic legacy, Vascular memory, Cardiovascular diseases

## Abstract

Based on the results of recent randomized, controlled clinical trials and analyses of their follow-up periods the concept of metabolic memory cannot be restricted to antihyperglycaemic treatment only, rather it can be extended to lipid-lowering and antihypertensive treatment and even life-style modification. This broadened concept can be designated as vascular memory. According to this new concept, not only immediate and short-term but long-term effects of the metabolic and cardiovascular risk milieu are of great importance. Consequently, early and intensive lifestyle interventions, treatment of hyperglycaemia, lipid abnormalities and hypertension can result in beneficial effects on cardiovascular outcomes even in the long run. On the contrary, failing in target-oriented treatment from early detection of abnormalities can be associated with life-threatening cardiovascular events subsequently. Additional experimental studies are needed to characterize the exact pathomechanism of vascular memory and further clinical trials are also essential to explore its real clinical significance.

## 

The concept of metabolic memory was first described among patients with type 1 diabetes in 2005, based on the results of the follow-up observation of the original cohort in the DCCT [1]. Although this term was already used in former experimental diabetes models and studies with isolated cells as early as the mid-1980s [2], the modern concept of metabolic memory emerged from the DCCT-EDIC. Reassuringly, late effect of previous antihyperglycaemic treatment was documented among patients with type 2 diabetes during the follow-up of the original cohort in the UKPDS [3]. The phenomenon was designated as metabolic legacy. Based on the results of recent randomized, controlled clinical trials and analyses of their follow-up periods it became obvious that the concept of metabolic memory cannot be restricted to antihyperglycaemic treatment only.

In this paper, clinical evidence concerning the late effect of antihyperglycaemic treatment is summarized. Additionally, the late effects of lipid-lowering and antihypertensive treatment as well as life-style modification are also reviewed. Taken together, results from recent clinical trials suggest that the original concept of metabolic memory can be defined in a much broader context.

## **Antihyperglycaemic treatment and its late effect in type 1 diabetes**

The DCCT was a multicenter, randomized, controlled clinical trial which compared intensive insulin therapy with conventional insulin regimens in patients with type 1 diabetes [4]. Originally, 1441 patients with type 1 diabetes were randomly assigned to either intensive or conventional insulin therapy and were followed for a mean of 6.5 years between 1983 and 1993. A significant difference in HbA1c values of the groups was found (mean values in patients with intensive treatment 7.4 % and that in patients with conventional treatment 9.0 %, p < 0.001). The risk of both development and progression of microvascular complications was significantly reduced by intensive insulin treatment. Nevertheless, due to the low incidence of cardiovascular events only a decreasing trend in risk of macrovascular complications was observed during the trial. The EDIC trial was a longitudinal observational study involving the cohort from the DCCT. The goal of this observational prospective evaluation was to assess the long-term effects of differences in prior treatment (intensive *versus* conventional insulin therapy during the DCCT) on the late development and progression of microvascular and macrovascular complications [5]. Although the absolute difference in HbA1c values between the groups was only 0.1 % (p = 0.38) at year 11 in the EDIC study, a consistent salutary effect of intensive insulin therapy was observed. Namely, by achieving glucose control as close to the nondiabetic range as safely possible, the risk of development and progression of micro- and macrovascular late complications was significantly reduced.

As for microvascular complications, the risk of retinopathy remained significantly reduced in the former group with intensive insulin treatment 4 years after completion of the original trial [6]. In addition, after 10 years in the DCCIT/EDIC follow up the rates of retinopathy proved to be lower than in the former group with conventional insulin treatment despite the converged HbA1c levels during the follow-up [7]. As for diabetic nephropathy, a reduction of microalbuminuria and clinical albuminuria was observed at 8 years of the follow-up. In addition, fewer cases with hypertension and transplantation due to diabetic nephropathy were recorded [8]. At a median follow-up of 13 years after persistent microalbuminuria, former intensive diabetes therapy proved to be associated with improved renal outcomes such as progression to microalbuminuria, impaired glomerular filtration rate, end-stage renal disease and regression to normoalbuminuria [9]. Over a median follow-up period of 22 years in the combined study (at a median follow-up of 16 years in the EDIC study) impairment of the glomerular filtration rate (GFR) developed in 24 participants assigned to intensive therapy and in 46 assigned to conventional therapy resulting in a 50 % risk reduction with intensive therapy (95 % CI 18‐69; p = 0.0006) [10]. As for neuropathy, signs of both somatic and autonomic neuropathy were less frequently observed in patients with early intensive glycaemic control in the DCCT/EDIC follow-up at 8 years [11]. Moreover, the benefits of former intensive insulin treatment persisted for 13‐14 years after the DCCT closeout and provide evidence of a durable effect of prior intensive treatment on both peripheral [12] and autonomic neuropathy [13].

As for cardiovascular complications, a beneficial effect of early intensive glycaemic control on surrogate endpoints such as progression of carotid intima-media thickness [14,15] or coronary artery calcification [16] was also shown during the EDIC follow-up at 6 and at 7‐9 years, respectively. A beneficial effect on cardiovascular events was also documented [1]. Namely, during the mean 17 years of follow-up, 46 cardiovascular events occurred in 31 patients who had received intensive treatment in the DCCT, as compared with 98 events in 52 patients who had received conventional treatment. Intensive treatment reduced the risk of any cardiovascular disease event by 42 % (95 % CI 9 to 63 %; p = 0.02) and the risk of nonfatal myocardial infarction, stroke, or death from cardiovascular disease by 57 % (95 % CI 12 to 79 %; p = 0.02).

**Figure 1:**
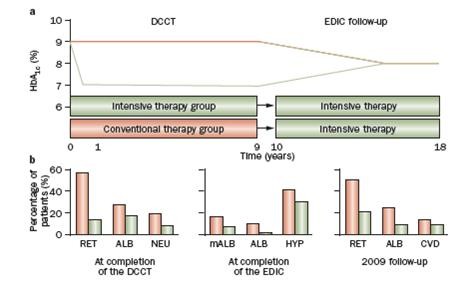
**The design of the DCCT-EDIC study and the development of late complications (From Nat Rev Endocrinol 2010; 6:665–675, with permission of the publisher [Nature Publishing Group])**. RET: retinopathy, ALB: albuminuria, NEU: neuropathy, mALB: microalbuminuria, HYP: hypertension, CVD: cardiovascular diseases.

Taken together, the DCCT/EDIC study demonstrated that an average period of 6.5 years of intensive insulin treatment had a long-term, sustained effect on the subsequent risk of late micro- and macrovascular complications (Figure 1). This phenomenon was designated by the authors as „metabolic memory” in 2005 [1].

## **Antihyperglycaemic treatment and its late effect in type 2 diabetes**

The UKPDS, one of the largest randomised clinical trials in diabetology, embracing 20 years of study (1977‐1997) followed by a further 10 years of post-trial monitoring, has radically altered our knowledge about the natural course and treatment of type 2 diabetes. The original trial with 4209 randomized patients documented that intensive glucose therapy *versus* conventional treatment (i.e. better glycaemic control) was associated with a reduced risk of clinically evident microvascular complications and a nonsignificant reduction in the relative risk of myocardial infarction in patients with newly diagnosed type 2 diabetes mellitus [17]. In the post-trial monitoring, 3277 patients were asked to attend annual UKPDS clinics for 5 years and later questionnaires were used to follow patients in years 6 to 10. Between-group differences in glycated hemoglobin levels were lost after at the first year after closeout. In the sulfonylurea/insulin group, relative reductions in risk persisted at 10 years for any diabetes-related end point and microvascular disease. In addition, as more events occurred, risk reductions for myocardial infarction and death from any cause emerged (Table 1). Similarly, a continued benefit after metformin therapy was also evident among overweight patients. In conclusion, a beneficial effect of better glycaemic control due to intensive treatment persisted over time despite the early loss of within-trial differences in glycated hemoglobin levels between the intensive-therapy group and the conventional-therapy group. The phenomenon was called as legacy effect of earlier glucose control [3].

**Table 1:** Legacy effect of earlier glucose control in the UKPDS (randomized phase of the trial completed in 1997, post-trial monitoring period [median 8.5 years] completed in 2007)

**Aggregate endpoint**	**1997**		**2007**	
	**RRR**	**p value**	**RRR**	**p value**
**Any diabetes related endpoint**	12 %	0.029	9 %	0.040
**Microvascular disease**	25 %	0.0099	24 %	0.001
**Myocardial infarction**	16 %	0.052	15 %	0.014
**All-cause mortality**	6 %	0.44	13 %	0.007

*RRR* relative risk reduction.

## **Lipid lowering treatment with statins and its late effect**

The legacy effect of lipid lowering treatment was documented by extended follow-up investigations of randomized controlled trials with statins (simvastatin, pravastatin, atorvastatin) supporting the idea of vascular memory.

The 4 S was one of the earliest randomized trials for assessing the effect of simvastatin in the secondary prevention of myocardial infarction [18]. The benefits of simvastatin in reducing mortality and cardiovascular events were clearly demonstrated in this study (median duration of follow-up 5.4 years). After completion, patients were followed for an additional 2 years (interim analysis), and later for 5 years during which open-label simvastatin treatment was provided for all patients [19,20]. It was found that simvastatin treatment for 5.4 years in a placebo-controlled trial, followed by open-label statin therapy for 5 years, was associated with a survival benefit over 10 years of follow-up compared with open-label statin therapy for the past 5 years only. It is of note, that during the post-trial period there were no differences in cardiovascular event rates in those originally assigned simvastatin or placebo but importantly, the survival benefit of patients allocated simvastatin compared with those allocated placebo that accrued during the double-blind trial period persisted during follow-up.

In the HPS, the efficacy and safety of lowering LDL-cholesterol with daily 40 mg simvastatin (versus placebo) were investigated [21]. During the in-trial period (mean follow-up 5.3 years), allocation to simvastatin yielded an average reduction in LDL cholesterol of 1.0 mmol/l and a proportional decrease in major vascular events of 23 % (95 % CI 19–28; p < 0.0001). During the post-trial period of 6.7 years (when statin use and lipid concentrations were similar in both groups), no further significant reductions were noted in either major vascular events (risk ratio 0.95 [95 % CI 0.89-1.02]) or vascular mortality (0.98 [95 % CI 0.90-1.07]). Nevertheless, the substantial reduction in vascular events among patients allocated simvastatin compared with those allocated placebo that was found in the randomized period of the trial persisted during observational follow-up after closeout of the original trial [22].

The WOSCOPS was designed to determine whether pravastatin in men with hypercholesterolaemia and no history of myocardial infarction reduced the combined incidence of nonfatal myocardial infarction and death from coronary heart disease [23]. After randomisation, patients were treated with either pravastatin or placebo for an average follow-up period of 4.9 years. In the double-blind phase of the trial, a 31 % relative risk reduction (p < 0.001) of combined endpoint was observed. The results of the long-term follow-up of the WOSCOPS were published later [24]. In the 10-year-long observational period, pravastatin was provided for all participants alive at the completion of the double blind phase. As for primary combined endpoint, the relative risk reduction favouring pravastatin was 18 % (p = 0.02) in the observational period while it was 27 % (p < 0.001) in the entire investigation (5 year double-blind phase + 10 year observational follow-up). Taken together, 5 years of treatment with pravastatin was associated with a significant reduction in coronary events for a subsequent 10 years in men with hypercholesterolaemia but without a history of myocardial infarction.

**Figure 2:**
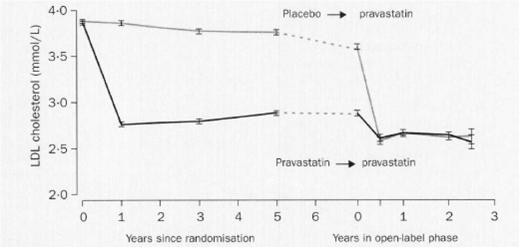
**LDL-cholesterol values (mean, 95 % confidence interval) in the LIPID trial.** A clear difference between pravastatin and placebo was observed in the double blind, randomized phase of the study (mean duration: 6 years) while the difference disappeared in the open-label, observational, post-trial follow up (duration: 2 years). (From Lancet 2002; 359:1379–1387, with permission).

**Figure 3:**
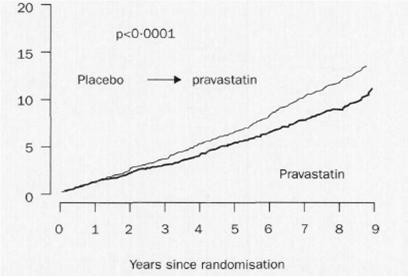
**Kaplan-Meier estimates illustrating the primary outcome (death from coronary artery disease [%]) in the LIPID trial.** In the double blind phase of the trial (mean duration: 6.0 years) a clear difference between pravastatin and placebo occurred which was maintained in the open, post-trial observational follow-up period (duration: 2.0 years) (From Lancet 2002; 359: 1379–1387, with permission).

The LIPID trial was designed to evaluate the effect of pravastatin (40 mg daily) versus placebo in 9014 patients (age 31‐75 years) with a history of myocardial infarction or hospitalization for unstable angina and initial plasma total cholesterol levels of 4.0 – 7.0 mmol/l [25]. The mean follow-up was 6.0 years. The relative risk reduction of death from coronary heart disease favouring pravastatin was 24 % (p < 0.001). After closing the double blind phase, the patients were followed for a further 2 years [26]. During this observational period, pravastatin was provided for participants treated formerly with placebo. In this period, no difference in plasma LDL-cholesterol values of the two groups was observed (Figure 2). Nevertheless, Kaplan-Meier curves of cardiovascular events and total mortality continuously diverged favouring the originally active *versus* control arms despite conversion of curves representing the changes of LDL-cholesterol values over time (Figure 3).

The ASCOT-LLA was a placebo-controlled randomized trial for evaluating the effects of atorvastatin 10 mg daily in the primary prevention of coronary heart disease in hypertensive subjects who had a total cholesterol level of ≤6.5 mmol/l. The trial was stopped prematurely after a median 3.3-year follow-up due to substantial benefits of atorvastatin (36 % relative risk reduction) on composite primary endpoint of cardiovascular events [27]. The results of extended observations 2.2 years after trial closure were published in 2008 [28] while those of the 11-year follow-up became available in 2011 [29]. At 2.2 years after the end of the ASCOT-LLA, the relative risk reduction in all endpoints remained essentially unchanged, although extensive crossovers from and to statin usage occurred. At 8 years after closure of ASCOT-LLA, all-cause mortality remained significantly lower in those originally assigned atorvastatin (HR 0.86, 95 % CI 0.76-0.98, p = 0.02). Cardiovascular deaths were fewer, but not significant (HR 0.89, 95 % CI 0.72-1.11, p = 0.32) and non-cardiovascular deaths were significantly lower (HR 0.85, 95 % CI 0.73-0.99, p = 0.03) in those formerly assigned atorvastatin attributed to a reduction in deaths due to infection and respiratory illness. The authors concluded that a legacy effect of those originally assigned atorvastatin might contribute to long-term benefits on all-cause mortality.

## **Treatment with antihypertensive drugs and its late effect**

The legacy effect of treatment with antihypertensive drugs was observed in some randomized, controlled trials with post-trial observational follow-up. Nevertheless, the beneficial late effect of tighter antihypertensive control was not observed in the UKPDS 10-year follow-up.

**Figure 4:**
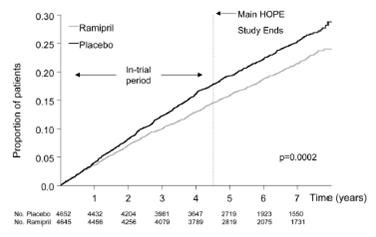
Kaplan-Meier estimates of the composite outcome of myocardial infarction, stroke, or cardiovascular death in the ramipril group and the placebo group of the HOPE trial and its extension (HOPE-TOO) (From Circulation 2005; 112:1339–1346, with permission).

In the HOPE study, the effect of ramipril *versus* placebo was assessed in patients who were at high risk for cardiovascular events but who did not have left ventricular dysfunction or heart failure [30]. A total of 9297 patients (age ≥55 years) were randomly assigned to receive ramipril (10 mg once per day) or matching placebo for a mean of 4.5 years. The primary outcome was a composite of myocardial infarction, stroke, or death from cardiovascular causes. A total of 651 patients who were assigned to receive ramipril (14.0 percent) reached the primary end point, as compared with 826 patients who were assigned to receive placebo (17.8 percent) (relative risk 0.78; 95 % confidence interval, 0.70 to 0.86; p < 0.001). The participants of the HOPE trial were followed up after terminating the original double blind phase for an additional 2.6 years in order to assess whether the benefits were maintained after trial cessation. During the extended follow-up (HOPE-TOO trial), in those who were event-free at the end of the HOPE study, there was a trend toward a further reduction in major cardiovascular events [31]. During the entire 7.2 years of follow-up, there was a significant risk reduction with ramipril for the primary composite outcome of myocardial infarction, stroke and cardiovascular death (relative risk reduction 17 %, p = 0.0002) (Figure 4).

In the TRACE study, patients with myocardial infarction and left ventricular systolic dysfunction (ejection fraction ≤35 %) were randomly assigned to receive oral trandolapril or placebo [32]. The follow-up was 24 to 50 months. The relative risk reduction of death in the trandolapril *versus* placebo group was 22 % (p = 0.001). The long-term benefit of trandolapril was evaluated over 10‐12 years of follow-up [33]. In the post-treatment period no difference was observed between groups (relative risk 1.03, p = 0.75) regarding all-cause mortality but for the entire follow-up, trandolapril significantly reduced the risk of death compared with placebo (relative risk reduction 11 %, p = 0.031). The authors concluded that in patients with left ventricular dysfunction, trandolapril given shortly after a myocardial infarction for 2–4 years has long-term benefits on mortality maintained for at least 10–12 years.

In the SOLVD trial, enalapril *versus* placebo was added to conventional therapy in patients with reduced (≤35 %) ejection fraction and a significant reduction in the incidence of heart failure and the rate of related hospitalizations was observed in a median duration of 37.4 months. In addition, there was a trend toward fewer deaths due to cardiovascular causes among the patients who received enalapril [34]. In order to establish whether the mortality reduction with enalapril was sustained, subsequent vital status was ascertained in 5165 individuals who were alive when the trial had been completed. The duration of the total follow-up was 12.1 years [35]. Beyond the original trial period, the survival curves continued to diverge in favour of the enalapril group for about 5 years, after which the curves started to converge. The authors concluded that the benefits of enalapril treatment continued to accrue beyond the end of the trial resulting in a sustained improvement in survival.

In the SYST-EUR study, elderly patients with isolated systolic hypertension after 2 years of randomized treatment with nitrendipine or placebo were followed for an additional 4 years with open-label antihypertensive treatment [36]. Patients who received early antihypertensive treatment, as compared with patients who initially received placebo, had a significantly greater reduction in the risk of stroke (28 %), cardiovascular complications (15 %) and total mortality (13 %). The benefits were even more pronounced in diabetic patients.

In the UKPDS with newly diagnosed type 2 diabetic patients, the effect of tight blood pressure control was also evaluated and, therefore, over a 4-year period 1148 patients with hypertension were randomized to tight or less-tight blood-pressure control regimens. Tight control was achieved by using ACE-inhibitor (captopril) or beta-blocker (atenolol) while less-tight control was maintained by antihypertensive treatment that excluded these agents. In the randomized trial, for tight as compared with the less-tight control of blood pressure, there were relative risk reductions of 24 % for any diabetes-related endpoint, 32 % for diabetes-related death, 44 % for stroke, and 37 % for microvascular disease [37]. During the 10-year post-interventional period, differences in blood pressure between the two groups during the trial disappeared within 2 years after termination of the trial and the benefits of previously improved blood pressure control were not sustained when between-group differences in blood pressure were lost [38].

## **Lifestyle modification and its late effect**

In the FDPS, 522 middle-aged, overweight subjects with impaired glucose tolerance were randomly assigned to either the intervention or the control group [39]. Each subject in the intervention group received individualized counselling aimed at reducing weight, total intake of fat, and intake of saturated fat and increasing intake of fiber and physical activity. The primary end point was the development of type 2 diabetes, the duration of follow-up was 3.2 years. The cumulative incidence of diabetes after four years was 11 % in the intervention group and 23 % in the control group. Overall, the risk of diabetes was 58 % lower (p < 0.001) in the intervention group than in the control group. After the active intervention period, participants who were still free of diabetes were further followed-up for a median of 3 years [40]. Beneficial changes achieved by participants in the intervention group were maintained after discontinuation of the intervention, and the corresponding incidence rates during the post-intervention follow-up were 4.6 and 7.2 per 100 person-years (p = 0.0401), indicating 36 % reduction in relative risk. Nevertheless, the active intervention period did not decrease the risk of cardiovascular morbidity and mortality during the first 10 years of follow-up [41].

In the China Da Qing Diabetes Prevention Study, 577 adult subjects with impaired glucose tolerance were randomly assigned to either the control group or to lifestyle intervention groups (diet, exercise, or both). Active intervention took place between 1986 and 1992 [42]. In 1992, after a 6-year intervention, participants were informed of the final results and asked to continue with normal medical care. In 2006, study participants were followed-up to assess the long-term effect of intervention [43]. Compared with control participants, those in the combined lifestyle intervention groups had a 51 % lower incidence of diabetes (hazard ratio 0.49; 95 % CI 0.33 ‐ 0.73) during the active intervention period and a 43 % lower incidence (hazard ratio 0.57; 95 % CI 0.41 ‐ 0.81) over the 20 year period.

In the 2.8 years of the DPP randomised clinical trial, diabetes incidence in high-risk adults was reduced by 58 % with intensive lifestyle intervention and by 31 % with metformin, compared with placebo [44]. During the 10-year follow-up since randomisation, incidences in the former placebo and metformin groups fell to equal those in the former lifestyle group, but the cumulative incidence of diabetes remained lowest in the lifestyle group. Accordingly, prevention or delay of diabetes with lifestyle intervention or metformin persisted for at least 10 years [45].

## **Multifactorial intervention and its late effect**

**Figure 5:**
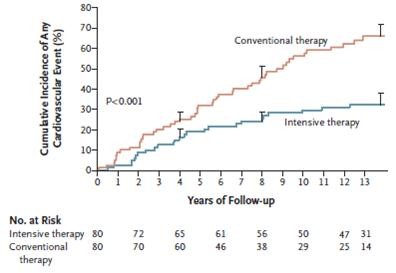
Cumulative incidence of cardiovascular events (including death from cardiovascular causes, nonfatal stroke, nonfatal myocardial infarction, coronary-artery bypass grafting, percutaneous coronary intervention, revascularization for peripheral atherosclerotic artery disease, and amputation) during the entire Steno-2 study (7.8 years of randomized, controlled trial and 5.5 years of open-label observational follow-up (From N Engl J Med 2008; 358:580–591, with permission).

The STENO-2 study, conducted over 7.8 years, recognized that the risk of cardiovascular events and death could be halved among patients with longstanding diabetes and microalbuminuria by intensive multifactorial treatment [46]. Patients from this cohort were subsequently followed for a mean of 5.5 years and results from this post-trial observation documented that intensive intervention had sustained beneficial effects with respect to vascular complications and death (Figure 5) [47].

## **Possible pathomechanism**

Although the exact pathomechanism of vascular memory is not clearly understood some elements of the pathophysiologic process have already been highlighted by experimental studies. Furthermore, explanations of the late effect of an earlier treatment have become available from clinical studies.

As for antihyperglycaemic treatment, increased formation of AGE could be a plausible cause of structural and functional changes occurring in the early metabolic environment but carrying implications for developing and progressing micro- and macrovascular complications in the long run [48-50]. In addition, altered mitochondrial function due to oxidative stress should also be considered [51]. Recently, increasing evidence suggests that epigenetic factors play a key role in the complex interplay between genes and the environment [52]. Thus, sustained hyperglycaemia can lead via methylation or histone acetylation to dysregulated epigenetic mechanisms that affect chromatin structure and gene expression [53-57]. Accordingly, the altered state of the epigenome might be the underlying mechanism contributing to the metabolic memory resulting in micro- and macrovascular dysfunction in diabetes even after achieving adequate glycaemic control. Nevertheless, it was found in a further experimental study that exposure to oscillating glucose was more deleterious than constant high glucose and induced a metabolic memory after glucose normalisation [58]. In clinical circumstances, the role of AGE formation was suggested as a rational basis for the phenomenon of metabolic memory in the DCCT cohort [59]. However, some other elements should also be considered. For example microalbuminuria, a well characterized cardiovascular risk factor, was less pronounced in the intensively treated subgroup during the entire DCCT-EDIC observation which could contribute to the more favourable cardiovascular outcome when subgroups with former intensive *versus* conventional treatment were compared [9]. Interestingly enough, re-analyzing the DCCT with respect to time-dependent memory effects of HbA1c revealed that the most current (in real time) HbA1c value was not the most important, but values from 2 to 3 years prior contributed the greatest risk to current progression of retinopathy [60]. Recently, links between microvascular dysfunction and subsequent macrovascular disease were supposed and microvascular structural changes were suggested to play a potential role in the metabolic memory [61].

Although late beneficial effect of early therapy with statins was documented in several lipid trials, the patomechanism remains obscure. Although the time of initiating treatment with statins and the dose of the respective statin are of great importance, the non-lipid lowering benefits of statins should also be considered. Generally, statins are linked to the inhibition of HMG-CoA reductase and a subsequent reduction in synthesis of isoprenoid intermediates by which a number of critical intracellular signalling processes are prevented leading to beneficial effect on subclinical inflammation and endothelial function. All these factors may have a role in vascular protection with statin therapy even long term [62,63].

As for treatment with antihypertensive drugs, it should be emphasized that no legacy effect in the UKPDS follow-up was observed [38]. Nevertheless, the absence of the legacy effect in the UKPDS hypertension sub-study is heavily debated [64]. It is of note that drugs (captopril or atenolol) used for achieving tight blood pressure control in this sub-study are not really used for long-term blood pressure control in current practice. In addition, the target blood pressure values in the UKPDS were far from those recommended in current guidelines [65]. The sub-study was not really powered to detect the late effect of antihypertensive treatment and the higher HbA1c values in the tight control group might mask the potential benefits during the follow-up. Consequently, no final conclusion can be drawn from the UKPDS hypertension sub-study regarding a legacy effect of antihypertensive treatment. It is of note, however, that results of other clinical trials with antihypertensive drugs (HOPE, TRACE, SOLVD) should be considered supportive for a legacy effect. In this respect it should be noted that RAAS dysregulation is heavily involved in triggering organ damage in patients with diabetes. Hyperglycaemia directly upregulates intracellular synthesis of angiotensin-II and high glucose stimulates angiotensinogen gene expression and cell hypertrophy. Ultimately, angiotensin-II activation leads to pathologic vessel remodelling [66]. In light of these observations, it seems obvious that blocking RAAS and consequently, interfering pathways involved in early organ damage may have late beneficial vascular effect. Conversely, delayed *versus* immediate start of antihypertensive treatment may have a deleterious effect on cardiovascular outcome [67].

Regarding life-style modification it is of great importance that weight reduction can result in improvement in both insulin resistance and beta-cell function. Accordingly, the incidence rate of diabetes mellitus in subjects originally recruited with impaired glucose tolerance proved to be lower in the intervention versus control groups during the randomized trial and even the post-trial period [68]. In this respect it is of note that some differences in patients’ weight between groups were maintained during the follow-up period of the FDPS [69]. Notably, results from studies with life-style modification indicated that incidence of type 2 diabetes rather than that of cardiovascular events decreased during the entire observation [41,43,45].

## **Summary and practical consequences of the vascular memory**

It is very likely that the term of metabolic memory observed in type 1 and type 2 diabetic patients with different antihyperglycaemic treatment [70,71] can be extended to antihypertensive and lipid-lowering treatment and even life-style modification. This broadened concept can be designated as vascular memory. Some years ago this broadened concept was considered as a hypothesis [72] but with more convincing follow-up data from recent randomized clinical trials it appears very likely that this phenomenon really exists.

According to the concept of the vascular memory, not only immediate and short-term but even long-term effects of the metabolic and cardiovascular risk milieu could be expected. Consequently, early and intensive treatment of hyperglycaemia, lipid abnormalities, and hypertension can result in beneficial effects on cardiovascular outcomes even in the long run (“good memory”). On the contrary, failing in target-oriented treatment from early detection of abnormalities can be associated with subsequent life-threatening cardiovascular events (“bad memory”) [73]. Similarly, late beneficial effect of life-style modification (versus regular care) on the incidence of type 2 diabetes was observed among subjects with impaired glucose tolerance. Importantly, ADDITION-Europe is the only randomized trial so far aiming to assess the effect of early intensive intervention of screen-detected patients with type 2 diabetes [74]. At the end of the 5-year prospective study a small, non-significant reduction in the incidence of cardiovascular events and death was observed.

Taken together, the “memory effect” is a new challenge for treatment aiming to reduce cardiometabolic risks and events. It is obvious that not only experimental studies but further clinical trials are needed to explore the exact pathomechanism and the particular clinical significance of the vascular memory.

## **Abbreviations**

ACE = Angiotensin-convertase enzyme; ADDITION-Europe = Anglo-Danish-Dutch Study of Intensive Treatment In People with Screen Detected Diabetes in Primary Care; AGE = Advanced glycation end products; ASCOT-LLA = Anglo-Scandinavian Cardiac Outcomes Trial Lipid Lowering Arm; DCCT = Diabetes Control and Complications Trial; DPP = Diabetes Prevention Program; EDIC = Epidemiology of Diabetes Interventions and Complications; FDPS = Finnish Diabetes Prevention Study; HOPE = Heart Outcomes Prevention Evaluation; HPS = Heart Protection Study; LIPID = Long-term Intervention with Pravastatin in Ischaemic Disease; RAAS = Renin-angiotensin-aldosterone system; SYST-EUR = Systolic Hypertension in Europe Trial; SOLVD = Studies of Left Ventricular Dysfunction; TRACE = Trandolapril Cardiac Evaluation; UKPDS = United Kingdom Prospective Diabetes Study; WOSCOPS = West of Scotland Coronary Prevention Study; 4 S = Scandinavian Simvastatin Survival Study.

## **Competing interests**

The author declares that he has no competing interests.

## **Author’s contribution**

GJ conceived, wrote and approved the final manuscript.
